# (*E*)-2′-[(3,5-Di-*tert*-butyl-2-hy­droxy­benzyl­idene)amino]-1,1′-binaphthalen-2-ol methanol monosolvate

**DOI:** 10.1107/S1600536811040116

**Published:** 2011-10-12

**Authors:** Dian He, Chong Li, Xiaohong Wang

**Affiliations:** a Institute of Medicinal Chemistry, School of Pharmacy, Lanzhou University, Lanzhou 730000, People’s Republic of China

## Abstract

The title compound, C_35_H_35_NO_2_·CH_4_O, was obtained by the reaction of *rac*-2-amino-2-hy­droxy-1,1-binaphthyl and 3,5-di-*tert*-butyl-2-hy­droxy­benzaldehyde in absolute methanol. In the Schiff base mol­ecule, the two naphthyl bicycles are twisted by 71.15 (5)°. One hy­droxy group is involved in intra­molecular O—H⋯N hydrogen bond, while the methanol solvent mol­ecule is linked to another hy­droxy group *via* an inter­molecular O—H⋯O hydrogen bond.

## Related literature

For applications of related compounds in stereo- and enanti­oselective reactions, see: Hu *et al.* (1999 ▶). For related structures, see: Yuan *et al.* (2002 ▶).
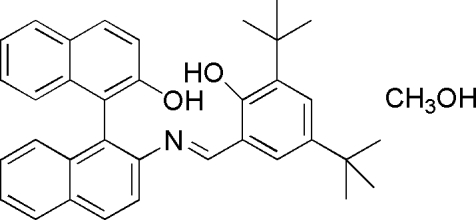


## Experimental

### 

#### Crystal data

C_35_H_35_NO_2_·CH_4_O
*M* *_r_* = 533.68
Monoclinic, 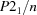

*a* = 8.8396 (3) Å
*b* = 12.2251 (5) Å
*c* = 28.3202 (11) Å
β = 95.018 (2)°
*V* = 3048.7 (2) Å^3^
*Z* = 4
Mo *K*α radiation
μ = 0.07 mm^−1^
*T* = 293 K
0.27 × 0.23 × 0.15 mm

#### Data collection

Bruker SMART APEX CCD area-detector diffractometer
Absorption correction: multi-scan (*SADABS*; Bruker, 2007 ▶) *T*_min_ = 0.981, *T*_max_ = 0.989
17039 measured reflections
6047 independent reflections
2734 reflections with *I* > 2σ(*I*)
*R*_int_ = 0.058

#### Refinement

*R*[*F* ^2^ > 2σ(*F* ^2^)] = 0.057
*wR*(*F* ^2^) = 0.177
*S* = 0.98
6047 reflections
371 parameters
H-atom parameters constrained
Δρ_max_ = 0.27 e Å^−3^
Δρ_min_ = −0.25 e Å^−3^

### 

Data collection: *APEX2* (Bruker, 2007 ▶); cell refinement: *SAINT* (Bruker, 2007 ▶); data reduction: *SAINT*; program(s) used to solve structure: *SHELXS97* (Sheldrick, 2008 ▶); program(s) used to refine structure: *SHELXL97* (Sheldrick, 2008 ▶); molecular graphics: *SHELXTL* (Sheldrick, 2008 ▶); software used to prepare material for publication: *SHELXTL*.

## Supplementary Material

Crystal structure: contains datablock(s) I, global. DOI: 10.1107/S1600536811040116/cv5152sup1.cif

Structure factors: contains datablock(s) I. DOI: 10.1107/S1600536811040116/cv5152Isup2.hkl

Supplementary material file. DOI: 10.1107/S1600536811040116/cv5152Isup3.cml

Additional supplementary materials:  crystallographic information; 3D view; checkCIF report

## Figures and Tables

**Table 1:** Hydrogen-bond geometry (Å, °)

*D*—H⋯*A*	*D*—H	H⋯*A*	*D*⋯*A*	*D*—H⋯*A*
O1—H1⋯O3	0.82	1.96	2.727 (4)	155
O2—H2⋯N1	0.82	1.85	2.582 (3)	147

**Fig. 1.**
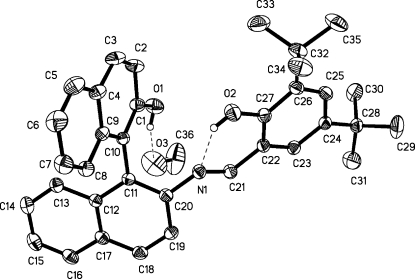
The molecular structure of (I) showing the atomic numbering and 30% probabilty displacment ellipsoids. Dashed lines denote hydrogen bonds. Hydrogen atoms, which are not involved in hydrogen bonding, have been excluded for clarity.

## supplementary crystallographic information

### Comment

The tridentate ligands containing Schiff base and hydroxybenzene group simultaneity have been widely used in organic catalytic reactions, such as stereoselective aldol addition reactions and enantioselective hetero-Diels-Alder reactions (Hu *et al.*, 1999; Yuan *et al.*, 2002). Herein, the synthesis and structure of a new tridentate ligand is reported.

In the title compound (Fig.1), the molecule adopts an E configuration at the C=N double bond. The dihedral angle between the phenyl ring (C22–C27, r.m.s. deviation 0.0056 Å) and two naphthyl rings (C1–C10, r.m.s. deviation 0.0231 Å, C11–C20, r.m.s. deviation 0.0196 Å) are 38.26 (8)° and 42.71 (9)°, respectively. Two naphthyl bicycles are twisted at 71.15 (5)°. One hydroxy group is involved in intramolecular O—H···N hydrogen bond (Table 1), while methanol solvent molecule is linked to another hydroxy group *via* intermolecular O—H···O hydrogen bond (Table 1, Fig. 1).

### Experimental

Rac-2-amino-2-hydroxy-1,1-binaphthyl (285 mg, 0.1 mmol) and 3,5-di-*tert*-butyl-2-hydroxybenzaldehyde (280 mg, 0.12 mmol) were stirred in absolute methanol (20 ml) and the mixture was heated to reflux for 24 h. The solvent was removed *in vacuo* and the crystals was isolated by recrystallization in methanol (420 mg, 84%). ^1^HNMR (CDCl_3_): 12.41 (s, 1 H), 8.60 (s, 1 H), 8.06 (d, J = 8.80 Hz, 1 H), 7.99 to 7.84 (m, 5 H), 7.59 to 7.06 (m, 8 H), 4.77 (s, 1 H), 1.47 (s, 9 H), 1.36 (s, 9 H); elemental analysis calcd (%) for C_36_H_39_NO_3_: C 81.02, H 7.37, N 2.62; found: C 81.37, H 7.04, N 2.87.

### Refinement

All H atoms were placed in calculated positions and refined using a riding model. The H atoms were situated into the idealized positions with the carrier atom-H distances = 0.93 Å for aryl and methylene group, 0.96 Å for the methyl and 0.82 Å for hydroxyl H atoms. The *U*_iso_ values were constrained to be 1.5*U*_eq_ of the carrier atom for the methyl H and hydroxyl H atoms and 1.2*U*_eq_ for the remaining H atoms.

### Crystal data

**Table:**

C_35_H_35_NO_2_·CH_4_O	*F*(000) = 1144
*M**_r_* = 533.68	*D*_x_ = 1.163 Mg m^−^^3^
Monoclinic, *P*2_1_/*n*	Mo *K*α radiation, λ = 0.71073 Å
Hall symbol: -P 2yn	Cell parameters from 17039 reflections
*a* = 8.8396 (3) Å	θ = 2.2–25.5°
*b* = 12.2251 (5) Å	µ = 0.07 mm^−^^1^
*c* = 28.3202 (11) Å	*T* = 293 K
β = 95.018 (2)°	Block, colourless
*V* = 3048.7 (2) Å^3^	0.27 × 0.23 × 0.15 mm
*Z* = 4	

### Data collection

**Table:**

Bruker SMART APEX CCD area-detector diffractometer	6047 independent reflections
Radiation source: fine-focus sealed tube	2734 reflections with *I* > 2σ(*I*)
graphite	*R*_int_ = 0.058
φ and ω scans	θ_max_ = 26.1°, θ_min_ = 1.4°
Absorption correction: multi-scan (*SADABS*; Bruker, 2007)	*h* = −9→10
*T*_min_ = 0.981, *T*_max_ = 0.989	*k* = −15→14
17039 measured reflections	*l* = −32→35

### Refinement

**Table:**

Refinement on *F*^2^	Secondary atom site location: difference Fourier map
Least-squares matrix: full	Hydrogen site location: inferred from neighbouring sites
*R*[*F*^2^ > 2σ(*F*^2^)] = 0.057	H-atom parameters constrained
*wR*(*F*^2^) = 0.177	*w* = 1/[σ^2^(*F*_o_^2^) + (0.0772*P*)^2^] where *P* = (*F*_o_^2^ + 2*F*_c_^2^)/3
*S* = 0.98	(Δ/σ)_max_ < 0.001
6047 reflections	Δρ_max_ = 0.27 e Å^−^^3^
371 parameters	Δρ_min_ = −0.25 e Å^−^^3^
0 restraints	Extinction correction: *SHELXL97* (Sheldrick, 2008), Fc^*^=kFc[1+0.001xFc^2^λ^3^/sin(2θ)]^-1/4^
Primary atom site location: structure-invariant direct methods	Extinction coefficient: 0.0041 (9)

### Special details

**Table:**

Geometry. All e.s.d.'s (except the e.s.d. in the dihedral angle between two l.s. planes) are estimated using the full covariance matrix. The cell e.s.d.'s are taken into account individually in the estimation of e.s.d.'s in distances, angles and torsion angles; correlations between e.s.d.'s in cell parameters are only used when they are defined by crystal symmetry. An approximate (isotropic) treatment of cell e.s.d.'s is used for estimating e.s.d.'s involving l.s. planes.
Refinement. Refinement of *F*^2^ against ALL reflections. The weighted *R*-factor *wR* and goodness of fit *S* are based on *F*^2^, conventional *R*-factors *R* are based on *F*, with *F* set to zero for negative *F*^2^. The threshold expression of *F*^2^ > σ(*F*^2^) is used only for calculating *R*-factors(gt) *etc*. and is not relevant to the choice of reflections for refinement. *R*-factors based on *F*^2^ are statistically about twice as large as those based on *F*, and *R*- factors based on ALL data will be even larger.

### Fractional atomic coordinates and isotropic or equivalent isotropic displacement parameters (Å^2^)

**Table:**

	*x*	*y*	*z*	*U*_iso_*/*U*_eq_	
C1	0.9594 (3)	0.5514 (2)	0.31889 (10)	0.0563 (7)	
C2	1.0468 (4)	0.6438 (2)	0.30968 (12)	0.0723 (9)	
H2A	1.0072	0.6965	0.2883	0.087*	
C3	1.1880 (4)	0.6569 (2)	0.33153 (12)	0.0735 (9)	
H3	1.2438	0.7190	0.3254	0.088*	
C4	1.2512 (3)	0.5780 (2)	0.36335 (11)	0.0607 (8)	
C5	1.3983 (4)	0.5896 (3)	0.38680 (13)	0.0814 (10)	
H5	1.4535	0.6527	0.3820	0.098*	
C6	1.4605 (4)	0.5111 (3)	0.41595 (13)	0.0891 (11)	
H6	1.5570	0.5206	0.4313	0.107*	
C7	1.3784 (3)	0.4157 (3)	0.42287 (11)	0.0769 (9)	
H7	1.4218	0.3609	0.4424	0.092*	
C8	1.2363 (3)	0.4019 (2)	0.40147 (10)	0.0579 (7)	
H8	1.1840	0.3376	0.4067	0.070*	
C9	1.1660 (3)	0.4827 (2)	0.37157 (9)	0.0487 (7)	
C10	1.0162 (3)	0.4699 (2)	0.34870 (8)	0.0457 (6)	
C11	0.9281 (3)	0.3678 (2)	0.35611 (9)	0.0449 (6)	
C12	0.8688 (3)	0.3457 (2)	0.40045 (9)	0.0468 (6)	
C13	0.8830 (3)	0.4200 (2)	0.43878 (9)	0.0550 (7)	
H13	0.9350	0.4854	0.4355	0.066*	
C14	0.8227 (3)	0.3984 (3)	0.48022 (10)	0.0679 (8)	
H14	0.8328	0.4492	0.5048	0.082*	
C15	0.7453 (3)	0.2999 (3)	0.48630 (11)	0.0747 (9)	
H15	0.7055	0.2848	0.5149	0.090*	
C16	0.7289 (3)	0.2276 (3)	0.45043 (11)	0.0685 (9)	
H16	0.6766	0.1628	0.4547	0.082*	
C17	0.7885 (3)	0.2467 (2)	0.40647 (10)	0.0555 (7)	
C18	0.7703 (3)	0.1724 (2)	0.36864 (10)	0.0624 (8)	
H18	0.7204	0.1065	0.3727	0.075*	
C19	0.8244 (3)	0.1949 (2)	0.32627 (10)	0.0581 (7)	
H19	0.8105	0.1448	0.3015	0.070*	
C20	0.9015 (3)	0.2939 (2)	0.31964 (9)	0.0493 (7)	
C21	0.8722 (3)	0.3009 (2)	0.23690 (9)	0.0551 (7)	
H21	0.7849	0.2593	0.2387	0.066*	
C22	0.9090 (3)	0.3373 (2)	0.19083 (9)	0.0500 (7)	
C23	0.8118 (3)	0.3108 (2)	0.15092 (9)	0.0548 (7)	
H23	0.7274	0.2672	0.1546	0.066*	
C24	0.8368 (3)	0.3470 (2)	0.10628 (9)	0.0547 (7)	
C25	0.9643 (3)	0.4147 (2)	0.10338 (10)	0.0593 (8)	
H25	0.9822	0.4416	0.0736	0.071*	
C26	1.0651 (3)	0.4444 (2)	0.14131 (10)	0.0564 (7)	
C27	1.0360 (3)	0.4039 (2)	0.18554 (10)	0.0530 (7)	
C28	0.7328 (3)	0.3183 (2)	0.06217 (9)	0.0631 (8)	
C29	0.8241 (4)	0.2717 (3)	0.02382 (11)	0.1129 (14)	
H29A	0.7565	0.2498	−0.0029	0.169*	
H29B	0.8809	0.2094	0.0360	0.169*	
H29C	0.8928	0.3264	0.0141	0.169*	
C30	0.6485 (4)	0.4195 (3)	0.04272 (12)	0.0931 (11)	
H30A	0.5909	0.4501	0.0667	0.140*	
H30B	0.5811	0.3996	0.0157	0.140*	
H30C	0.7203	0.4726	0.0335	0.140*	
C31	0.6155 (5)	0.2328 (3)	0.07330 (12)	0.1196 (15)	
H31A	0.5506	0.2625	0.0956	0.179*	
H31B	0.6663	0.1691	0.0866	0.179*	
H31C	0.5555	0.2131	0.0447	0.179*	
C32	1.2008 (3)	0.5213 (3)	0.13554 (12)	0.0735 (9)	
C33	1.1867 (4)	0.6246 (3)	0.16564 (14)	0.1059 (13)	
H33A	1.1914	0.6049	0.1985	0.159*	
H33B	1.0914	0.6598	0.1566	0.159*	
H33C	1.2683	0.6738	0.1606	0.159*	
C34	1.3488 (4)	0.4612 (3)	0.15019 (14)	0.0992 (12)	
H34A	1.3586	0.3996	0.1296	0.149*	
H34B	1.3479	0.4362	0.1823	0.149*	
H34C	1.4329	0.5099	0.1478	0.149*	
C35	1.2075 (4)	0.5579 (3)	0.08416 (13)	0.1151 (14)	
H35A	1.2956	0.6028	0.0818	0.173*	
H35B	1.1178	0.5991	0.0742	0.173*	
H35C	1.2132	0.4948	0.0642	0.173*	
C36	0.4695 (6)	0.4186 (5)	0.28155 (19)	0.191 (3)	
H36A	0.3997	0.3614	0.2878	0.286*	
H36B	0.5049	0.4078	0.2508	0.286*	
H36C	0.4191	0.4881	0.2824	0.286*	
N1	0.9532 (2)	0.32263 (17)	0.27522 (8)	0.0525 (6)	
O1	0.8170 (2)	0.55086 (17)	0.29590 (8)	0.0789 (6)	
H1	0.7662	0.5027	0.3071	0.118*	
O2	1.1314 (2)	0.42890 (18)	0.22408 (7)	0.0712 (6)	
H2	1.0928	0.4106	0.2482	0.107*	
O3	0.5808 (3)	0.4169 (3)	0.31260 (13)	0.1635 (14)	
H3A	0.6335	0.3631	0.3085	0.245*	

### Atomic displacement parameters (Å^2^)

**Table:**

	*U*^11^	*U*^22^	*U*^33^	*U*^12^	*U*^13^	*U*^23^
C1	0.0537 (17)	0.0523 (18)	0.0620 (18)	0.0033 (14)	0.0002 (15)	0.0066 (14)
C2	0.081 (2)	0.0495 (19)	0.087 (2)	0.0057 (17)	0.0124 (19)	0.0192 (16)
C3	0.071 (2)	0.0451 (18)	0.107 (3)	−0.0111 (16)	0.021 (2)	0.0046 (18)
C4	0.0540 (17)	0.0513 (18)	0.077 (2)	−0.0075 (14)	0.0073 (16)	−0.0044 (15)
C5	0.060 (2)	0.074 (2)	0.109 (3)	−0.0216 (18)	0.003 (2)	−0.009 (2)
C6	0.057 (2)	0.099 (3)	0.107 (3)	−0.016 (2)	−0.0191 (19)	−0.002 (2)
C7	0.059 (2)	0.088 (3)	0.080 (2)	−0.0021 (18)	−0.0120 (17)	0.0078 (18)
C8	0.0509 (17)	0.0583 (19)	0.0633 (18)	−0.0026 (14)	−0.0027 (14)	0.0045 (15)
C9	0.0456 (15)	0.0468 (16)	0.0533 (16)	−0.0040 (13)	0.0021 (13)	−0.0047 (13)
C10	0.0477 (15)	0.0428 (15)	0.0460 (15)	−0.0011 (12)	0.0009 (12)	0.0007 (12)
C11	0.0417 (14)	0.0455 (16)	0.0466 (16)	−0.0035 (12)	−0.0007 (12)	0.0048 (12)
C12	0.0408 (14)	0.0513 (17)	0.0475 (16)	−0.0004 (12)	−0.0008 (12)	0.0030 (13)
C13	0.0499 (16)	0.0613 (19)	0.0529 (17)	0.0045 (13)	−0.0003 (14)	0.0019 (14)
C14	0.0648 (19)	0.087 (2)	0.0516 (19)	0.0082 (18)	0.0049 (15)	−0.0023 (17)
C15	0.0625 (19)	0.108 (3)	0.055 (2)	0.002 (2)	0.0097 (16)	0.011 (2)
C16	0.0583 (18)	0.082 (2)	0.066 (2)	−0.0108 (16)	0.0063 (16)	0.0202 (18)
C17	0.0488 (16)	0.0619 (19)	0.0559 (18)	−0.0023 (14)	0.0050 (14)	0.0086 (15)
C18	0.0630 (18)	0.0571 (19)	0.067 (2)	−0.0154 (14)	0.0043 (16)	0.0075 (16)
C19	0.0618 (17)	0.0526 (18)	0.0590 (18)	−0.0144 (14)	0.0007 (15)	−0.0048 (14)
C20	0.0449 (15)	0.0528 (17)	0.0496 (17)	−0.0079 (13)	0.0005 (13)	0.0038 (13)
C21	0.0525 (16)	0.0607 (18)	0.0519 (18)	−0.0117 (13)	0.0042 (14)	−0.0014 (14)
C22	0.0513 (16)	0.0528 (17)	0.0463 (16)	−0.0027 (13)	0.0068 (13)	−0.0012 (13)
C23	0.0548 (16)	0.0554 (18)	0.0543 (18)	−0.0055 (13)	0.0052 (14)	−0.0052 (14)
C24	0.0666 (18)	0.0494 (17)	0.0480 (17)	0.0075 (14)	0.0047 (15)	−0.0012 (13)
C25	0.0702 (19)	0.0560 (18)	0.0535 (18)	0.0081 (15)	0.0152 (16)	0.0101 (14)
C26	0.0594 (18)	0.0520 (17)	0.0595 (19)	0.0022 (14)	0.0146 (16)	0.0063 (14)
C27	0.0512 (16)	0.0571 (18)	0.0503 (17)	−0.0005 (14)	0.0016 (14)	−0.0008 (14)
C28	0.082 (2)	0.0585 (19)	0.0480 (17)	0.0094 (16)	0.0004 (16)	−0.0023 (14)
C29	0.140 (3)	0.132 (4)	0.064 (2)	0.047 (3)	−0.004 (2)	−0.028 (2)
C30	0.103 (3)	0.091 (3)	0.081 (2)	0.027 (2)	−0.019 (2)	−0.006 (2)
C31	0.161 (4)	0.117 (3)	0.072 (2)	−0.058 (3)	−0.041 (3)	0.000 (2)
C32	0.071 (2)	0.071 (2)	0.081 (2)	−0.0109 (17)	0.0187 (17)	0.0156 (18)
C33	0.111 (3)	0.074 (3)	0.135 (3)	−0.026 (2)	0.022 (3)	0.002 (2)
C34	0.064 (2)	0.109 (3)	0.128 (3)	−0.010 (2)	0.028 (2)	0.030 (2)
C35	0.121 (3)	0.122 (3)	0.105 (3)	−0.032 (2)	0.032 (2)	0.041 (3)
C36	0.102 (4)	0.295 (8)	0.164 (5)	0.058 (4)	−0.055 (4)	−0.080 (5)
N1	0.0515 (13)	0.0579 (15)	0.0479 (14)	−0.0072 (11)	0.0022 (11)	−0.0018 (11)
O1	0.0666 (13)	0.0788 (17)	0.0877 (16)	0.0068 (11)	−0.0133 (12)	0.0230 (12)
O2	0.0639 (13)	0.0872 (15)	0.0622 (13)	−0.0221 (10)	0.0045 (11)	0.0025 (12)
O3	0.0793 (19)	0.220 (4)	0.187 (3)	0.003 (2)	−0.010 (2)	0.039 (3)

### Geometric parameters (Å, °)

**Table:**

C1—O1	1.366 (3)	C22—C27	1.405 (3)
C1—C10	1.372 (3)	C23—C24	1.375 (3)
C1—C2	1.406 (4)	C23—H23	0.9300
C2—C3	1.353 (4)	C24—C25	1.406 (4)
C2—H2A	0.9300	C24—C28	1.526 (4)
C3—C4	1.403 (4)	C25—C26	1.383 (4)
C3—H3	0.9300	C25—H25	0.9300
C4—C5	1.414 (4)	C26—C27	1.392 (3)
C4—C9	1.417 (4)	C26—C32	1.543 (4)
C5—C6	1.351 (4)	C27—O2	1.355 (3)
C5—H5	0.9300	C28—C29	1.520 (4)
C6—C7	1.396 (4)	C28—C30	1.522 (4)
C6—H6	0.9300	C28—C31	1.524 (4)
C7—C8	1.357 (4)	C29—H29A	0.9600
C7—H7	0.9300	C29—H29B	0.9600
C8—C9	1.410 (3)	C29—H29C	0.9600
C8—H8	0.9300	C30—H30A	0.9600
C9—C10	1.431 (3)	C30—H30B	0.9600
C10—C11	1.495 (3)	C30—H30C	0.9600
C11—C20	1.377 (3)	C31—H31A	0.9600
C11—C12	1.428 (3)	C31—H31B	0.9600
C12—C13	1.412 (3)	C31—H31C	0.9600
C12—C17	1.421 (3)	C32—C34	1.526 (4)
C13—C14	1.357 (4)	C32—C35	1.528 (4)
C13—H13	0.9300	C32—C33	1.535 (5)
C14—C15	1.403 (4)	C33—H33A	0.9600
C14—H14	0.9300	C33—H33B	0.9600
C15—C16	1.345 (4)	C33—H33C	0.9600
C15—H15	0.9300	C34—H34A	0.9600
C16—C17	1.413 (4)	C34—H34B	0.9600
C16—H16	0.9300	C34—H34C	0.9600
C17—C18	1.403 (4)	C35—H35A	0.9600
C18—C19	1.358 (3)	C35—H35B	0.9600
C18—H18	0.9300	C35—H35C	0.9600
C19—C20	1.410 (3)	C36—O3	1.261 (4)
C19—H19	0.9300	C36—H36A	0.9600
C20—N1	1.420 (3)	C36—H36B	0.9600
C21—N1	1.275 (3)	C36—H36C	0.9600
C21—C22	1.442 (3)	O1—H1	0.8200
C21—H21	0.9300	O2—H2	0.8200
C22—C23	1.396 (3)	O3—H3A	0.8200
			
O1—C1—C10	124.2 (2)	C23—C24—C28	123.0 (3)
O1—C1—C2	114.5 (3)	C25—C24—C28	121.1 (3)
C10—C1—C2	121.3 (3)	C26—C25—C24	125.0 (3)
C3—C2—C1	120.7 (3)	C26—C25—H25	117.5
C3—C2—H2A	119.7	C24—C25—H25	117.5
C1—C2—H2A	119.7	C25—C26—C27	116.6 (2)
C2—C3—C4	120.8 (3)	C25—C26—C32	122.1 (3)
C2—C3—H3	119.6	C27—C26—C32	121.3 (3)
C4—C3—H3	119.6	O2—C27—C26	119.6 (2)
C3—C4—C5	122.1 (3)	O2—C27—C22	119.5 (2)
C3—C4—C9	118.9 (3)	C26—C27—C22	121.0 (2)
C5—C4—C9	119.0 (3)	C29—C28—C30	108.7 (3)
C6—C5—C4	121.6 (3)	C29—C28—C31	107.7 (3)
C6—C5—H5	119.2	C30—C28—C31	108.2 (3)
C4—C5—H5	119.2	C29—C28—C24	110.6 (2)
C5—C6—C7	119.4 (3)	C30—C28—C24	110.5 (2)
C5—C6—H6	120.3	C31—C28—C24	111.1 (2)
C7—C6—H6	120.3	C28—C29—H29A	109.5
C8—C7—C6	120.9 (3)	C28—C29—H29B	109.5
C8—C7—H7	119.6	H29A—C29—H29B	109.5
C6—C7—H7	119.6	C28—C29—H29C	109.5
C7—C8—C9	121.5 (3)	H29A—C29—H29C	109.5
C7—C8—H8	119.2	H29B—C29—H29C	109.5
C9—C8—H8	119.2	C28—C30—H30A	109.5
C8—C9—C4	117.5 (2)	C28—C30—H30B	109.5
C8—C9—C10	122.5 (2)	H30A—C30—H30B	109.5
C4—C9—C10	119.9 (2)	C28—C30—H30C	109.5
C1—C10—C9	118.3 (2)	H30A—C30—H30C	109.5
C1—C10—C11	121.7 (2)	H30B—C30—H30C	109.5
C9—C10—C11	120.0 (2)	C28—C31—H31A	109.5
C20—C11—C12	118.9 (2)	C28—C31—H31B	109.5
C20—C11—C10	120.0 (2)	H31A—C31—H31B	109.5
C12—C11—C10	121.1 (2)	C28—C31—H31C	109.5
C13—C12—C17	117.9 (2)	H31A—C31—H31C	109.5
C13—C12—C11	122.6 (2)	H31B—C31—H31C	109.5
C17—C12—C11	119.5 (2)	C34—C32—C35	107.2 (3)
C14—C13—C12	121.6 (3)	C34—C32—C33	110.8 (3)
C14—C13—H13	119.2	C35—C32—C33	107.4 (3)
C12—C13—H13	119.2	C34—C32—C26	109.5 (2)
C13—C14—C15	120.4 (3)	C35—C32—C26	112.0 (3)
C13—C14—H14	119.8	C33—C32—C26	109.9 (2)
C15—C14—H14	119.8	C32—C33—H33A	109.5
C16—C15—C14	119.5 (3)	C32—C33—H33B	109.5
C16—C15—H15	120.2	H33A—C33—H33B	109.5
C14—C15—H15	120.2	C32—C33—H33C	109.5
C15—C16—C17	122.2 (3)	H33A—C33—H33C	109.5
C15—C16—H16	118.9	H33B—C33—H33C	109.5
C17—C16—H16	118.9	C32—C34—H34A	109.5
C18—C17—C16	122.6 (3)	C32—C34—H34B	109.5
C18—C17—C12	119.0 (2)	H34A—C34—H34B	109.5
C16—C17—C12	118.4 (3)	C32—C34—H34C	109.5
C19—C18—C17	121.2 (3)	H34A—C34—H34C	109.5
C19—C18—H18	119.4	H34B—C34—H34C	109.5
C17—C18—H18	119.4	C32—C35—H35A	109.5
C18—C19—C20	120.2 (3)	C32—C35—H35B	109.5
C18—C19—H19	119.9	H35A—C35—H35B	109.5
C20—C19—H19	119.9	C32—C35—H35C	109.5
C11—C20—C19	121.1 (2)	H35A—C35—H35C	109.5
C11—C20—N1	117.1 (2)	H35B—C35—H35C	109.5
C19—C20—N1	121.8 (2)	O3—C36—H36A	109.5
N1—C21—C22	123.5 (2)	O3—C36—H36B	109.5
N1—C21—H21	118.3	H36A—C36—H36B	109.5
C22—C21—H21	118.3	O3—C36—H36C	109.5
C23—C22—C27	119.2 (2)	H36A—C36—H36C	109.5
C23—C22—C21	119.3 (2)	H36B—C36—H36C	109.5
C27—C22—C21	121.4 (2)	C21—N1—C20	120.2 (2)
C24—C23—C22	122.2 (2)	C1—O1—H1	109.5
C24—C23—H23	118.9	C27—O2—H2	109.5
C22—C23—H23	118.9	C36—O3—H3A	109.5
C23—C24—C25	115.9 (2)		
			
O1—C1—C2—C3	177.5 (3)	C16—C17—C18—C19	177.7 (3)
C10—C1—C2—C3	−2.6 (4)	C12—C17—C18—C19	−1.8 (4)
C1—C2—C3—C4	0.9 (5)	C17—C18—C19—C20	0.5 (4)
C2—C3—C4—C5	−179.8 (3)	C12—C11—C20—C19	−3.4 (4)
C2—C3—C4—C9	1.3 (4)	C10—C11—C20—C19	177.3 (2)
C3—C4—C5—C6	−177.4 (3)	C12—C11—C20—N1	175.7 (2)
C9—C4—C5—C6	1.4 (5)	C10—C11—C20—N1	−3.6 (3)
C4—C5—C6—C7	0.6 (5)	C18—C19—C20—C11	2.2 (4)
C5—C6—C7—C8	−1.3 (5)	C18—C19—C20—N1	−176.9 (2)
C6—C7—C8—C9	0.0 (4)	N1—C21—C22—C23	−179.0 (3)
C7—C8—C9—C4	2.0 (4)	N1—C21—C22—C27	−2.4 (4)
C7—C8—C9—C10	−179.9 (3)	C27—C22—C23—C24	0.5 (4)
C3—C4—C9—C8	176.2 (2)	C21—C22—C23—C24	177.1 (2)
C5—C4—C9—C8	−2.7 (4)	C22—C23—C24—C25	−1.5 (4)
C3—C4—C9—C10	−2.0 (4)	C22—C23—C24—C28	179.5 (2)
C5—C4—C9—C10	179.2 (2)	C23—C24—C25—C26	1.4 (4)
O1—C1—C10—C9	−178.2 (2)	C28—C24—C25—C26	−179.6 (2)
C2—C1—C10—C9	1.9 (4)	C24—C25—C26—C27	−0.3 (4)
O1—C1—C10—C11	4.2 (4)	C24—C25—C26—C32	−178.6 (3)
C2—C1—C10—C11	−175.7 (2)	C25—C26—C27—O2	179.2 (2)
C8—C9—C10—C1	−177.7 (2)	C32—C26—C27—O2	−2.5 (4)
C4—C9—C10—C1	0.4 (4)	C25—C26—C27—C22	−0.8 (4)
C8—C9—C10—C11	−0.1 (4)	C32—C26—C27—C22	177.6 (2)
C4—C9—C10—C11	178.0 (2)	C23—C22—C27—O2	−179.3 (2)
C1—C10—C11—C20	68.0 (3)	C21—C22—C27—O2	4.2 (4)
C9—C10—C11—C20	−109.6 (3)	C23—C22—C27—C26	0.7 (4)
C1—C10—C11—C12	−111.3 (3)	C21—C22—C27—C26	−175.9 (2)
C9—C10—C11—C12	71.2 (3)	C23—C24—C28—C29	−127.9 (3)
C20—C11—C12—C13	−176.2 (2)	C25—C24—C28—C29	53.1 (4)
C10—C11—C12—C13	3.0 (4)	C23—C24—C28—C30	111.7 (3)
C20—C11—C12—C17	2.1 (3)	C25—C24—C28—C30	−67.2 (3)
C10—C11—C12—C17	−178.6 (2)	C23—C24—C28—C31	−8.4 (4)
C17—C12—C13—C14	0.1 (4)	C25—C24—C28—C31	172.7 (3)
C11—C12—C13—C14	178.4 (2)	C25—C26—C32—C34	−118.9 (3)
C12—C13—C14—C15	0.7 (4)	C27—C26—C32—C34	62.9 (4)
C13—C14—C15—C16	−1.1 (4)	C25—C26—C32—C35	−0.1 (4)
C14—C15—C16—C17	0.5 (5)	C27—C26—C32—C35	−178.3 (3)
C15—C16—C17—C18	−179.2 (3)	C25—C26—C32—C33	119.2 (3)
C15—C16—C17—C12	0.3 (4)	C27—C26—C32—C33	−59.0 (4)
C13—C12—C17—C18	178.9 (2)	C22—C21—N1—C20	173.3 (2)
C11—C12—C17—C18	0.5 (4)	C11—C20—N1—C21	−140.4 (3)
C13—C12—C17—C16	−0.6 (3)	C19—C20—N1—C21	38.7 (4)
C11—C12—C17—C16	−179.0 (2)		

### Hydrogen-bond geometry (Å, °)

**Table:**

*D*—H···*A*	*D*—H	H···*A*	*D*···*A*	*D*—H···*A*
O1—H1···O3	0.82	1.96	2.727 (4)	155.
O2—H2···N1	0.82	1.85	2.582 (3)	147.
